# Motivational Profiles in Unemployment: A Self-Determination Perspective

**DOI:** 10.3389/fpubh.2022.870073

**Published:** 2022-04-29

**Authors:** Leoni van der Vaart, Anja Van den Broeck, Sebastiaan Rothmann, Hans De Witte

**Affiliations:** ^1^Optentia Research Unit, North-West University, Vanderbijlpark, South Africa; ^2^Department of Work and Organization Studies, Faculty of Economics and Business, KU Leuven, Brussels, Belgium; ^3^Work, Organizational and Personnel Psychology, Occupational and Organizational Psychology and Professional Learning, Faculty of Psychology and Educational Sciences, KU Leuven, Leuven, Belgium

**Keywords:** attitudes, behavior, experiences, latent profile analysis, motivation, person-centered

## Abstract

In general, being unemployed has negative implications for the individual and the mental health of the public as a collective. One way to escape this situation is to search for a job. However, following self-determination theory (SDT), unemployed people's different reasons (i.e., their motivation) for engaging in a job search influence their well-being, attitudes, and behaviors for better or worse. Some research has already supported the associations between different types of motivation and these outcomes, but less is known about how these types of motivation simultaneously associate with these outcomes. The current study addressed this issue by studying how different motivational profiles had different implications for the affective experiences, commitment to employment, and job search behavior of the unemployed. Latent profile analysis, among 865 unemployed individuals from previously disadvantaged communities in South Africa, highlighted four distinct motivational profiles: motivated, ambivalent, amotivated, and unmotivated. The motivated reported some good well-being (i.e., positive experiences) and economic outcomes (i.e., employment commitment and job search), but these came at a cost (i.e., more negative experiences). The same went for the ambivalent, but to a lesser extent. Being unmotivated seemed to have the opposite effect in that it came with psychological benefits, but with economic costs, as these individuals might withdraw from the labor market. This also applied to the amotivated, although they experienced less psychological benefit than their unmotivated counterparts. The findings made several contributions to SDT and unemployment research and could help tailor interventions and policies for particular types of unemployed people.

## Introduction

Unemployment is a challenging issue around the globe, and specifically in South Africa, even more so due to the recent pandemic (1). It not only has important economic and societal costs but it is also a psychological burden for most unemployed individuals (2, 3). Therefore, most unemployed individuals are motivated to search for a job to escape this situation. However, such searching for a job can be fueled by different motivations, which matter for their experiences, commitment, and degree of job search.

Self-determination theory (SDT) argues that not only the amount, but also the type, of motivation matters for maintaining job search efforts and likely also has profound implications for the well-being of the unemployed and their attitudes to employment (4). While not being motivated (i.e., amotivation) or being compelled (i.e., controlled motivation) to look for a job are important predictors of negative experiences, attitudes, and behavior, searching for a job because you truly value the activity (i.e., identified regulation) may alleviate the burden of unemployment and may increase chances of being re-employed (5–7).

Although studies examining the relations between unemployed people's different types of motivation and their functioning exist, they used a variable-centered approach (5–8) which does not take into account that behavior is multi-motivated (9). Hence, they don't provide insights into how these different reasons may jointly influence how people feel, think, and behave. However, studies in other domains (such as work) have shown that the combination of different types and levels of motivation leads to various motivational profiles with different implications for people's experiences, attitudes, and behavior (10, 11). Therefore, studying the relations between motivation and unemployed individuals' functioning only from a variable-centered approach may lack ecological validity and hamper the true understanding of the implications of the different types of motivation (12, 13) for the unemployed.

Therefore, we conducted person-oriented research to understand better the association between the unemployed's motivation and their affective experiences, commitment to employment, and job search behavior. Despite its added value, such a person-oriented motivational perspective has not yet been applied in the unemployment context. Extrapolating the findings from another context may not be straightforward, as several researchers have advanced that different profiles may emerge in different contexts (14, 15). As the unemployment context is a pressuring context where the outcome (employment) is often not within control and amotivation reigns, we could expect the emergence of profiles that are particular to this context, with their own particular implications for individuals' well-being, attitudes, and behavior.

In short, the current study thus aimed to examine the motivational functioning of the unemployed by exploring their profiles based on their different types of motivation (as mentioned in SDT) to look for a job and the associated affective experiences, attitudes, and job search behavior. Studying the associations of these combinations in different domains may teach us more about the prevalence, nature, and consequences of these types of motivation with important implications for science and practice.

## Literature Overview

### Motivational Profiles Within Self-Determination Theory

Studies have applied a person-centered approach to the types of motivation of the SDT in a variety of contexts (i.e., education, sport, and work) (11, 14, 16). In the literature, the nature of these profiles differs from profiles consisting of low to high levels of the more controlled types of motivation^1^ in combination with low to high levels of the more autonomous types of motivation (11, 13). In general, studies have supported the assumptions of the SDT, showing that people with different profiles varying in the amount (i.e., having high, moderate, or low scores on each motivational regulation) and types (i.e., more autonomous vs. more controlled profiles) of motivation reported differences in well-being, attitudes, and behavior.

However, the contexts of these studies influenced the nature of the profiles that emerged (14, 15, 17). More specifically, a review of the literature illustrated that studies in the work context reported more truly autonomous profiles than studies in the educational (especially among high school students) and sport (especially among elite athletes) contexts, in which more controlled profiles emerged. Given the unique nature of the unemployment context, it was, thus, necessary to investigate profiles in this context to understand how the different types of motivation combined and the consequences of this. In such a pressurizing and discouraging context, one would most likely find controlled profiles rather than autonomous profiles. Amotivation might also be more prominent than in other contexts, resulting in amotivated profiles being more prevalent among the unemployed than in other contexts. Interestingly, we envisioned that amotivation might, furthermore, co-present with high levels of motivation—in those motivated to search, yet feeling that (re-)employment was not attainable because of personal and structural barriers.

The current study took an exploratory approach in identifying the number and configurations of profiles, considering the variations in profiles in previous studies in different contexts and the absence of evidence in the unemployment context. Given that previous research typically identified between three (18, 19) and six (20, 21) profiles, we thought it likely that a similar number of profiles would be identified using latent profile analysis (LPA), and we expected the established profiles to differ in both amount (i.e., levels) and quality (i.e., shape) of motivation. We, thus, posed the following broad hypothesis:

Hypothesis 1: different motivational profiles exist among the unemployed, differing in the amount and quality of motivation.

We expected these profiles to differ in outcomes, which would further attest to their construct validity and practical relevance (22). In the current study, we included affective experiences, employment commitment, and job search intensity to evaluate the concurrent validity of the motivational profiles. These outcome variables have important implications for the well-being and (re-)employment of the unemployed (3).

### Experiences, Attitudes, and Behavior as a Function of Profile Membership

The unemployed may have many negative affective experiences such as little social contact with people outside their immediate family, loss of their social identity, having no shared purpose, and the absence of a daily time structure and participation in regular activities (23). However, some unemployed may also have positive experiences, such as enjoying the extra time they spend with significant others or feeling more relaxed (24, 25). To fully understand how unemployed individuals with divergent motivational profiles differ in their experience of unemployment, we included both negative and positive experiences. Perceiving the job search as futile (i.e., amotivated profiles) or as a series of “musts” and “should” (i.e., controlled profiles) could potentially deprive the unemployed even more of the benefits they would have enjoyed as employees and prevent them from making the most of their available time. In contrast, the opposite should hold for unemployed individuals with more autonomous profiles (4). Therefore, we hypothesized as follows:

Hypothesis 2: profiles with higher levels of amotivation should report the highest levels of negative experiences and the lowest levels of positive experiences, followed by profiles with higher levels of controlled motivation. In contrast, autonomously motivated profiles should report the lowest levels of negative experiences and the highest levels of positive experiences.

To understand the attitudes of the unemployed to the labor market, we studied commitment to employment (26), which is defined as “the extent to which a person wants to be engaged in paid work” [(27), p. 130]. As one goes through the motions of a job search not seeing value in employment (5), amotivated profiles should associate with a lower commitment to employment. Also, searching for a job because one has to could alienate those with a controlled profile from the value of employment and should have negative implications (5, 6). Autonomous profiles should associate with a higher value attached to employment, as performing activities that are volitional and consistent with the self elicit positive attitudes (21, 28). We, therefore, posed this hypothesis:

Hypothesis 3: profiles with higher levels of amotivation should report the lowest levels of commitment, followed by profiles with higher levels of controlled motivation. In contrast, autonomously motivated profiles should report the highest levels of commitment.

Finally, we studied job search behavior, which is the most important problem-focused coping strategy of the unemployed (29). It is captured by the frequency with which individuals engage in a particular job search activity within a specific time frame (3). Lacking the motivation to act and persist (i.e., amotivated profiles) (5) is likely associated with low levels of job search, even more so than feeling pressurized and experiencing stress while engaging in the job search (i.e., controlled profiles) (5, 6). In contrast, unemployed individuals who can identify with looking for a job (i.e., autonomous motivation) will most likely invest more energy in searching for a job (5–7).

Hypothesis 4: profiles with higher levels of amotivation should then report the lowest levels of job search, followed by profiles with controlled motivation. In contrast, autonomously motivated profiles should report the highest levels of job search.

## Methods

### Participants and Procedure

Participants were recruited from two informal settlements in the Gauteng province of SA: Boipatong and Orange Farm. Since 1955, Boipatong has housed the former employees of a manufacturing company, but the turbulence in the world steel market has led to the organization reducing its labor force (30) drastically. Orange Farm, a much younger township, was developed by former farmworkers and has attracted many unemployed or retrenched farmworkers since 1988. It has become the largest and most populous informal settlement in SA. Most of its (unskilled) residents live in shacks, with inadequate infrastructure to access the community (31). We used convenience (i.e., recruiting unemployed participants roaming the streets and door-to-door) and volunteer sampling (i.e., advertisements in community newspapers and on community radio stations) to obtain a heterogeneous sample of the unemployed. Permission was obtained from the Humanities and Health Research Ethics Committee (HHREC) of the North-West University (NWU-HS-2016-0002) and the Social and Societal Ethics Committee (SMEC) of KU Leuven (G-2016 01 452). During the first visit, an information letter was given to prospective participants, in the language of their choice; the information was also explained verbally to the prospective participants to enhance understanding. The participants were allowed 24 h to decide whether they wanted to participate, after which the fieldworker returned to their homes with the consent letter and the questionnaire. Due to the lower levels of education of the participants, fieldworkers assisted them in filling out the questionnaires by reading the questions out loud (i.e., structured interviews) and translating questions from English to one of the local languages when necessary. A back-translation judgmental design was employed to determine the equivalence of translated questionnaires (32).

The final sample consisted of 867 participants from Boipatong (54.20%) and Orange Farm (45.80%). Slightly more females (54.30%) than males (45.70%) participated. Almost all participants (99.20%) were black, and most spoke either Sesotho (46.80%) or isiZulu (28.50%). The majority did not complete (58.70%) or only completed (38.70%) secondary education. On average, they were 32 years old (*SD* = 10.46), and most had been unemployed for more than 2 years (64.90%). Quite a number of them were single (79.30%), and more than half of them were living with parents or grandparents (or other family members) (50.50%), with no other income in the household from either employment or self-employment (50.10%). On average, they had two family members who were financially dependent on them (*SD* = 2), with two-thirds (66.10%) reporting social assistance received by others in the household. Only 22.50% received grants themselves. The sample was mostly characteristic of the unemployed in SA.

### Measures

#### Background Characteristics

Several variables were included that are commonly associated with motivation and/or experiences, attitudes, and behavior in an unemployment context: gender, age, educational level, marital status, living situation, area (township), unemployment duration, employment history, social assistance (self or others) or another form of income earned by others in the household, and the number of individuals financially dependent on the unemployed participant.

#### Motivation

Twenty-six items adapted^2^ from the Job Search Self-Regulation Questionnaire (5) tapped into individuals' motivation to search. Respondents were asked to rate the extent to which they agreed with statements reflecting personal amotivation (e.g., “I do not look for a job because I do not know how to start searching for a job”), structural amotivation (e.g., “I do not look for a job because there are no jobs available”), external regulation (e.g., “I look for a job because I feel pressure from others to do so”), introjected regulation (e.g., “I look for a job because I feel ashamed of being unemployed”), and identified regulation (e.g., “I look for a job because work is personally important to me”). The rating was done on a three-point Likert-type scale, ranging from 1 (*disagree*) to 3 (*agree*). A three-point scale was employed, as it is less cognitively demanding and is usually more appropriate when researching those with lower levels of education.

#### Experiences

Participants' positive (e.g., relaxed) and negative (e.g., lonely) affective experiences were measured by means of 16 items derived from the Experience of Unemployment Questionnaire (EUQ) (34) that had previously been used in the SA context (26). These positive and negative affective experiences were rated on a three-point frequency scale, ranging from 0 (*never*) to 2 (*often*).

#### Commitment

The importance participants attached to employment was measured by seven items based on the Employment Commitment Scale of Warr et al. (27), which had also been used in the SA context by De Witte et al. (26). Participants needed to indicate the extent to which they agreed with a range of statements (e.g., “I find it important to have work”) on a three-point Likert-type scale, ranging from 1 (*disagree*) to 3 (*agree*).

#### Job Search Behavior

Job search intensity was measured by asking how many times participants had performed any of the seven different job search behaviors [e.g., “Searched for advertisements on social media (e.g., Facebook, LinkedIn)” or “Submitted job applications”]. Participants needed to reflect on these behaviors using a five-point frequency scale, ranging from 0 (*never*) to 4 (*10 times or more*) (34). The EUQ items used by De Witte et al. (34) were adapted by adding two items to accommodate more recent job search methods (i.e., social media).

### Statistical Analysis

Mplus 8.6 (35) was used for data analyses. First, independent cluster modeling (ICM) using confirmatory factor analysis (CFA) and exploratory structural equation modeling (ESEM) were performed using the mean- and variance-adjusted weighted least squares (WLSMV) estimator to evaluate the preliminary measurement models. This approach was chosen because other approaches to evaluate the construct-relevant multidimensionality of constructs have been criticized. Specifically, exploratory factor analysis (EFA) is deemed inappropriate when validating instruments with an a priori factor structure. The ICM approach, used as part of CFA, is too strict as it assumes that items only load onto their own (target) factors. This assumption is deemed unrealistic because indicators (or items) are rarely uniquely related to only one factor, especially when constructs overlap theoretically (36), as is the case here. ESEM allows researchers to work from an a priori defined factor structure, yet relaxes the strict assumptions underlying CFA (36). It provides more exact estimates of factor correlations (37) and is therefore better suited to test motivation's continuum structure (38, 39).

Due to the cross-sectional nature of the data and the adaptations made to some of the measuring instruments, several measurement models were evaluated to determine the most optimal factor-analytic solution. Testing competing measurement models further provides evidence for the construct validity of the instruments. The fit of these models was assessed based on recommendations by Kline (40), Morin et al. (36) and Van Zyl and Ten Klooster (41). The reliabilities of the scales were calculated using the ordinal version of Cronbach's alpha reliability coefficient, which is appropriate for estimating the reliability of variables at an ordinal level (42).

Second, LPA was performed on the factor scores saved from preliminary measurement models, a practice that is more common in recent applications of mixture models (22, 43). This procedure controls for measurement error by giving more weight to items with lower levels of measurement error, thus reducing bias to which scale scores are prone (44). LPA is a model-based approach to cluster individuals or cases into groups (i.e., latent profiles) based on their responses to observed continuous variables (45). LPA was preferred for this study because of the possibility of using objective, statistical indices and tests to decide the optimal number of profiles [see (46, 47) for a full overview of the benefits]. Formal statistical procedures, based on Wang and Wang (48), were used to determine the optimal number of profiles. We used the indices recommended by Tofighi and Enders (49) to compare the fit of the models with different numbers of profiles. Simulation research has looked at the performance of these various indicators to facilitate decision-making regarding the optimal number of latent profiles [see (13) for a brief overview]. Simulation studies have demonstrated that the Bayesian information criterion (BIC) and the bootstrap likelihood ratio test (BLRT) outperform the other indices (50, 51), and these were used in the current research to inform decision-making.

Finally, we used the Bolck-Croon-Hagenaars (BCH) method [see (52) for a detailed description] to compare the levels of negative experiences, positive experiences, employment commitment, and job search behavior across the profiles as another means to validate the profiles, rather than defining them. BCH is the preferred method for continuous covariates (53), which is the case with factor scores.

## Results

### Preliminary Measurement Models

First, the factor analytic models for motivation were specified without the outcome variables to retain independence between the profiles and the covariates. Several competing ICM-CFA and ESEM models were estimated (see the Supplementary Material for more information). Following the fit statistics reported in Supplementary Table S1, the ESEM solution fitted the data best: χ^2^ = 354.49, *df* = 205, *p* < 0.001; root mean square error of approximation (RMSEA) = 0.029 [0.024, 0.034], *p* = 1.00; comparative fit index (CFI) = 0.99; Tucker–Lewis index (TLI) = 0.98; standardized root mean squared residual (SRMR) = 0.03.

Second, the dependent well-being, attitude, and behavioral variables were modeled (separate from the motivation variables to retain independence) in line with theory and previous empirical work (8): negative experiences (10 items), positive experiences (six items), the importance of work (seven items), and job search behavior (seven items). The revised model^3^ (Model 1a) had acceptable fit on most fit statistics (M1: χ^2^ = 1305.91, *df* = 399, *p* < 0.001; RMSEA = 0.051 [0.048, 0.054], *p* = 0.26; CFI = 0.87; TLI = 0.85; SRMR = 0.08; M1a: χ^2^ = 1041.66, *df* = 369, *p* < 0.001; RMSEA = 0.046 [.043, 0.049], *p* = 0.98; CFI = 0.90; TLI = 0.89; SRMR = 0.07). Last, a set-ESEM model was specified (consisting of an ESEM-within-CFA model plus the covariates) to enable reporting of the correlations between the motivation variables and the covariates (χ^2^ = 2468.10, *df* = 1310, *p* < 0.001; RMSEA = 0.035 [.033, 0.037], *p* = 1.00; CFI = 0.93; TLI = 0.92; SRMR = 0.07). Means, standard deviations, reliability coefficients, and correlations for all variables used in the present study are reported in Table 1.

**Table 1:** Means, standard deviations, reliability estimates, and correlations.

	**M**	***SD***	**1**	**2**	**3**	**4**	**5**	**6**	**7**	**8**	**9**
1. Structural amotivation	0.00 (1.79)	0.83 (0.71)	(0.95)								
2. Personal amotivation	0.04 (1.47)	0.85 (0.51)	0.70^***^	(0.94)							
3. Extrinsic regulation	−0.01 (2.38)	0.80 (0.46)	0.08	−0.03	(0.77)						
4. Introjected regulation	−0.02 (2.20)	0.82 (0.60)	0.23^***^	0.16^*^	0.58^***^	(0.88)					
5. Identified regulation	−0.11 (2.85)	0.73 (0.35)	−0.04	−0.25^**^	0.27^**^	0.12	(0.90)				
6. Negative experiences	−0.01 (1.35)	0.57 (0.44)	0.15^***^	−0.01	0.46^***^	0.45^***^	0.32^***^	(0.89)			
7. Positive experiences	0.01 (0.98)	0.41 (0.42)	−0.20^***^	0.06	0.08	0.07	−0.23^***^	−0.02	(0.72)		
8. Commitment	−0.04 (2.74)	0.57 (0.30)	0.02	−0.17^**^	0.52^***^	0.44^***^	0.60^***^	0.50^***^	−0.01	(0.91)	
9. Job search	−0.00 (2.11)	0.56 (0.93)	−0.12^**^	−0.07	0.07	0.01	0.13^*^	0.18^***^	0.02	0.09	(0.86)

*M, mean; SD, standard deviation; means and standard deviations estimated from scale scores indicated in brackets; ordinal version of Cronbach's alpha reliability coefficients provided in brackets on the diagonal;*

* *p < 0.05,*

** *p < 0.01,*

*** *p < 0.001*.

### Latent Profile Analysis

Next, the optimal number of motivational profiles was determined by estimating models—based on the ESEM factor scores—with increasing numbers of latent profiles based on the theoretical meaning and the statistical adequacy of the solutions. An exploratory approach (specifying as many profiles as the data allowed) was warranted, as the study set out to investigate profiles in a new context. Models with one to six profiles were specified. Table 2 shows the fit indices for the models.

**Table 2:** Comparison of profile models.

**Model**	**Log-likelihood**	**#fp**	**Scaling**	**AIC**	**BIC**	**ABIC**	**Entropy**	**LMR**	**ALMR**	**BLRT**	**Smallest class proportion**
1-profile	−5,199.55	10	0.86	10,419.11	10,466.76	10,435.00	–	–	–	–	–
2-profile	−4,764.32	16	1.00	9,560.65	9,636.89	9,586.08	0.88	0.00	0.00	0.00	38.45%
3-profile	−4,568.64	22	1.41	9,181.27	9,286.11	9,216.24	0.88	0.07	0.07	0.00	35.90%
4-profile	−4,415.16	28	1.14	8,886.31	9,019.74	8,930.82	0.85	0.00	0.00	0.00	16.72%
5-profile	−4,319.66	34	1.61	8,707.32	8,869.34	8,761.36	0.86	0.47	0.47	0.00	7.26%
6-profile	−4,227.70	40	1.25	8,535.41	8,726.01	8,598.98	0.84	0.00	0.00	0.00	5.98%

*#fp, number of free parameters; AIC, Akaike information criterion; BIC, Bayesian information criterion; ABIC, sample-size adjusted BIC; LMR, p-value associated with the Lo-Mendell-Rubin likelihood ratio test; ALMR, p-value associated with the adjusted Lo-Mendell-Rubin likelihood ratio test; BLRT, p-value associated with the bootstrap likelihood ratio test*.

**Figure 1:**
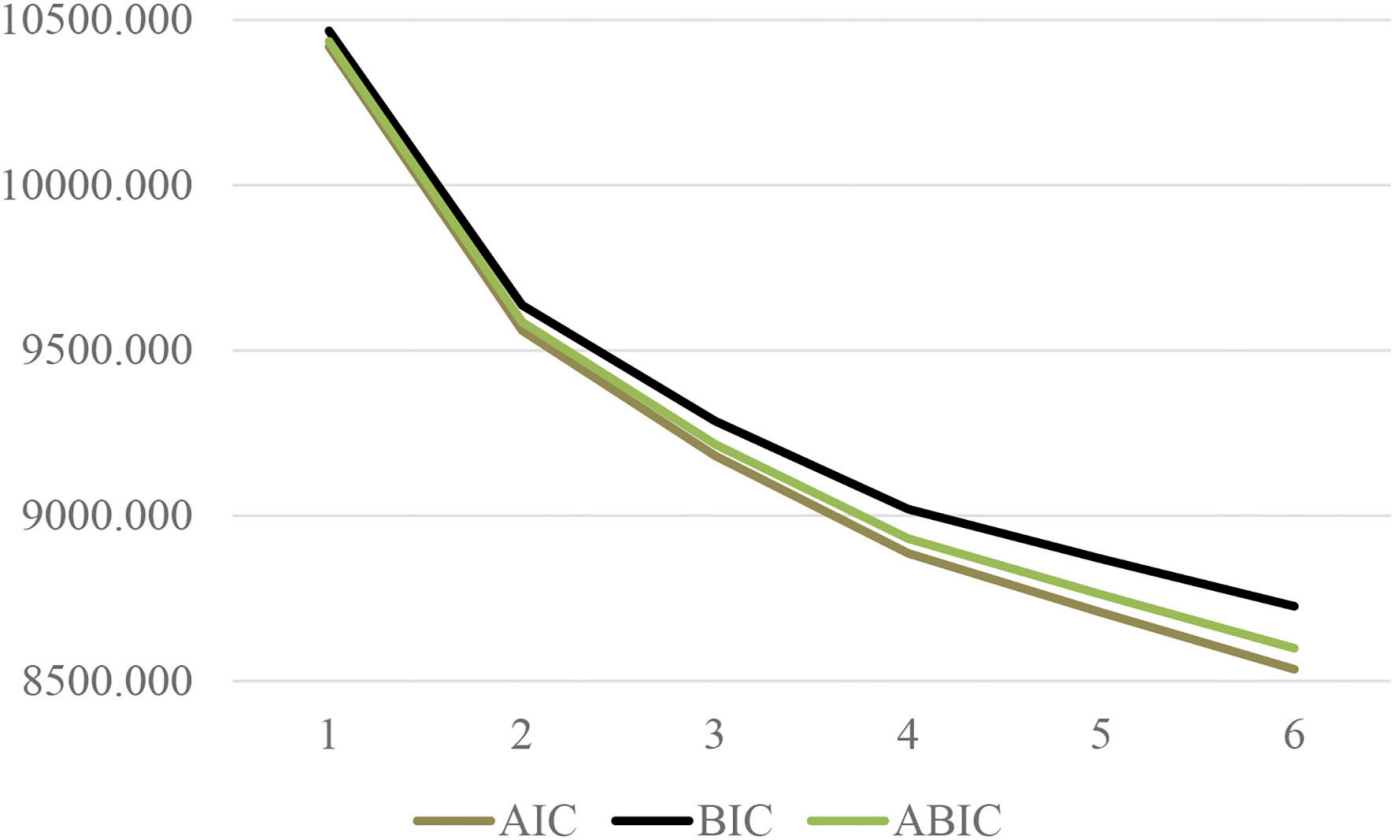
Elbow plot for the information criteria.

Examination of the plots indicated that adding a fourth profile resulted in the addition of a qualitatively and quantitatively different profile, compared to the model with three profiles. Adding a fifth profile resulted in a division of an existing profile (Profile 2) into two smaller profiles (with similar levels of all types of motivation), but did not add anything theoretically meaningful (i.e., there were no shape differences between these two profiles). Therefore, from a theoretical point of view, the solution representing four motivational profiles was preferred. The AIC, BIC, and ABIC values continued to decrease, and the BLRT remained statistically significant (*p* < 0.001). As this might be influenced by sample size (54), we examined the elbow plots (see Figure 1) to see whether indicators might improve without reaching a minimum (13). These plots supported a meaningful improvement from the three- to the four-profile solution, indicating that a plateau was not reached. Based on all of these observations, the more parsimonious four-profile model was preferred. The solution was theoretically interpretable and yielded adequate profiling (i.e., reliable classification of cases into the profiles); the posterior class membership probabilities were all above 0.86 [exceeding 0.70 as recommended by (55)], and the entropy value (of 0.85) exceeded 0.80, translating into high classification certainty (56).

**Figure 2:**
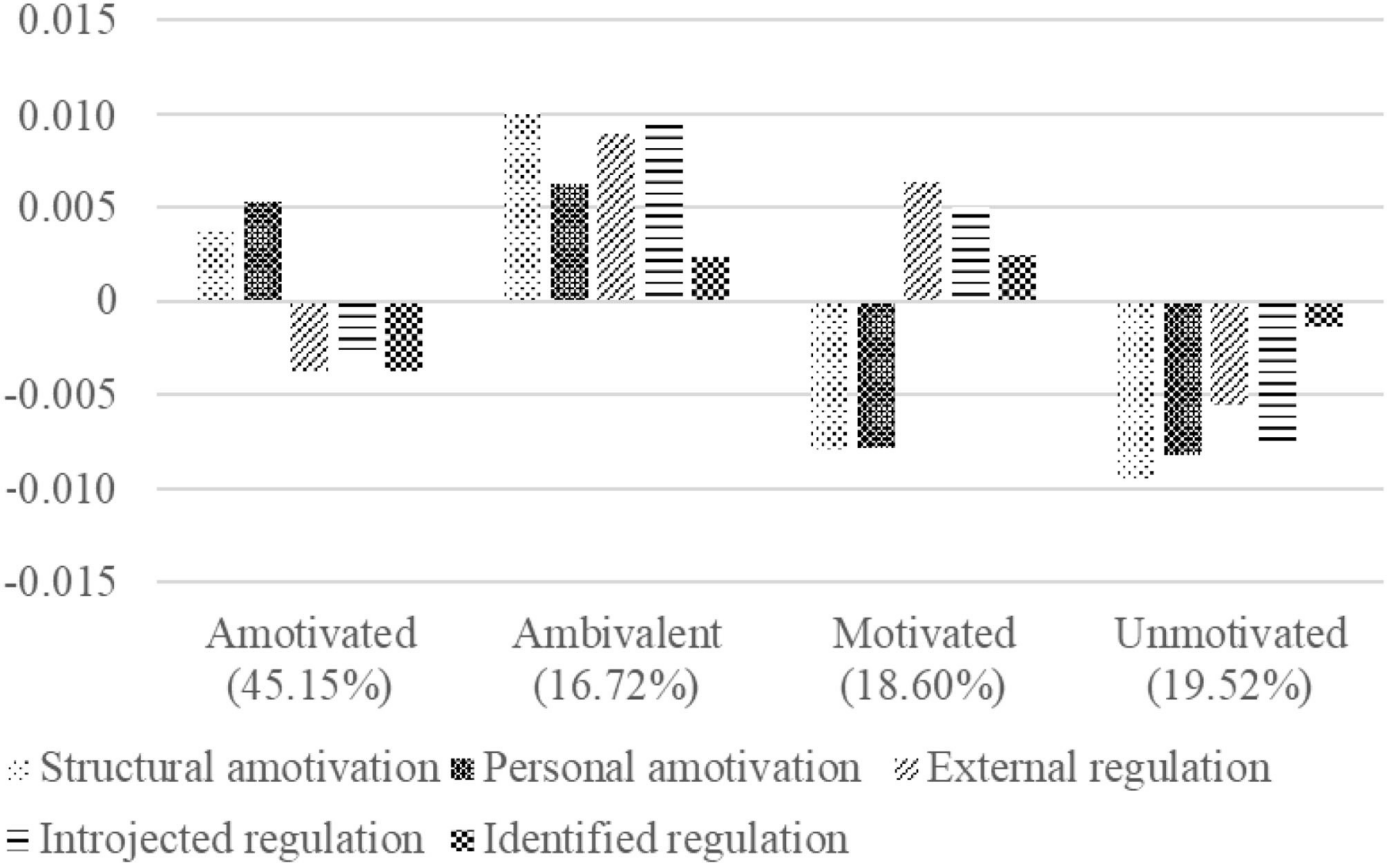
Description of the motivational profiles. Standardized factor score plot with an average of 0 and a standard deviation of 1.

As displayed in Figure 2, the four profiles differed in levels and types of motivational regulation. This result provided support for Hypothesis 1. As also can be understood from the upper half of Table 3, Profile 1 characterized the amotivated unemployed (45.15% of the sample), presenting high levels of amotivation and average to low levels of external, introjected, and identified regulation. Profile 2 characterized the ambivalent unemployed (16.72% of the sample), presenting high to very high levels of all types of motivational regulation. Profile 3 characterized the motivated unemployed (18.60% of the sample), presenting very low levels of amotivation and high levels of external, introjected, and identified regulation. Profile 4 characterized the unmotivated unemployed (19.52% of the sample), presenting very low levels of amotivation and low to very low levels of external, introjected, and identified regulation.

**Table 3:** Mean-level differences between retained motivational groups.

	**Amotivated**	**Ambivalent**	**Motivated**	**Unmotivated**
Structural amotivation	0.37^b^ (2.10)	1.00^a^ (2.69)	−0.79^c^ (1.10)	−0.95^d^ (1.03)
Personal amotivation	0.53^a^ (1.70)	0.63^a^ (1.81)	−0.79^b^ (1.06)	−0.82^b^ (1.04)
External regulation	−0.38^c^ (2.18)	0.89^a^ (2.85)	0.63^b^ (2.74)	−0.56^d^ (2.09)
Introject regulation	−0.26^c^ (2.05)	0.94^a^ (2.84)	0.50^b^ (2.63)	−0.79^d^ (1.61)
Identified regulation	−0.37^c^ (2.76)	0.24^a^ (2.99)	0.25^a^ (2.98)	−0.13^b^ (2.85)
Negative experiences	−0.14^b^ (1.28)	0.34^a^ (1.59)	0.32^a^ (1.55)	−0.34^c^ (1.13)
Positive experiences	−0.01^b^ (0.96)	−0.11^c^ (0.89)	0.17^a^ (1.12)	−0.01^b^ (0.98)
Commitment	−0.25^b^ (2.64)	0.36^a^ (2.90)	0.28^a^ (2.85)	−0.20^b^ (2.71)
Job search	−0.05^b^ (2.04)	−0.05^b^ (2.00)	0.16^a^ (2.32)	−0.01^b^ (2.17)

*Indicators estimated from scaled scores indicated in brackets; within rows, means with different letters are significantly different from one another*.

We compared the four profiles on participants' background characteristics (see Supplementary Table S5). Chi-square (χ^2^) tests indicated that the four groups differed regarding age [χ^2^ (*df* = 9, *n* = 859) = 59.37, *p* < 0.001], education [χ^2^ (*df* = 6, *n* = 861) = 30.58, *p* < 0.001], marital status [χ^2^ (*df* = 3, *n* = 861) = 19.53, *p* < 0.001], living situation [χ^2^ (*df* = 15, *n* = 861) = 53.12, *p* < 0.001], area [χ^2^ (*df* = 3, *n* = 867) = 44.67, *p* < 0.001], and grants (claimed by others in the household) [χ^2^ (*df* = 9, *n* = 817) = 26.49, *p* < 0.001]. Cramer's V provides a measure of the strength of the association between the categorical variables (57). Cohen's (58) guidelines were used to determine the magnitude of the practical effect sizes: small (0.10), medium (0.30) and large (0.50). These associations were generally small: 0.15 (age), 0.13 (education), 0.15 (marital status), 0.14 (living situation), 0.23 (township), and.10 (grants—others).

### Experiences, Attitudes, and Behavior as a Function of Profile Membership

**Figure 3:**
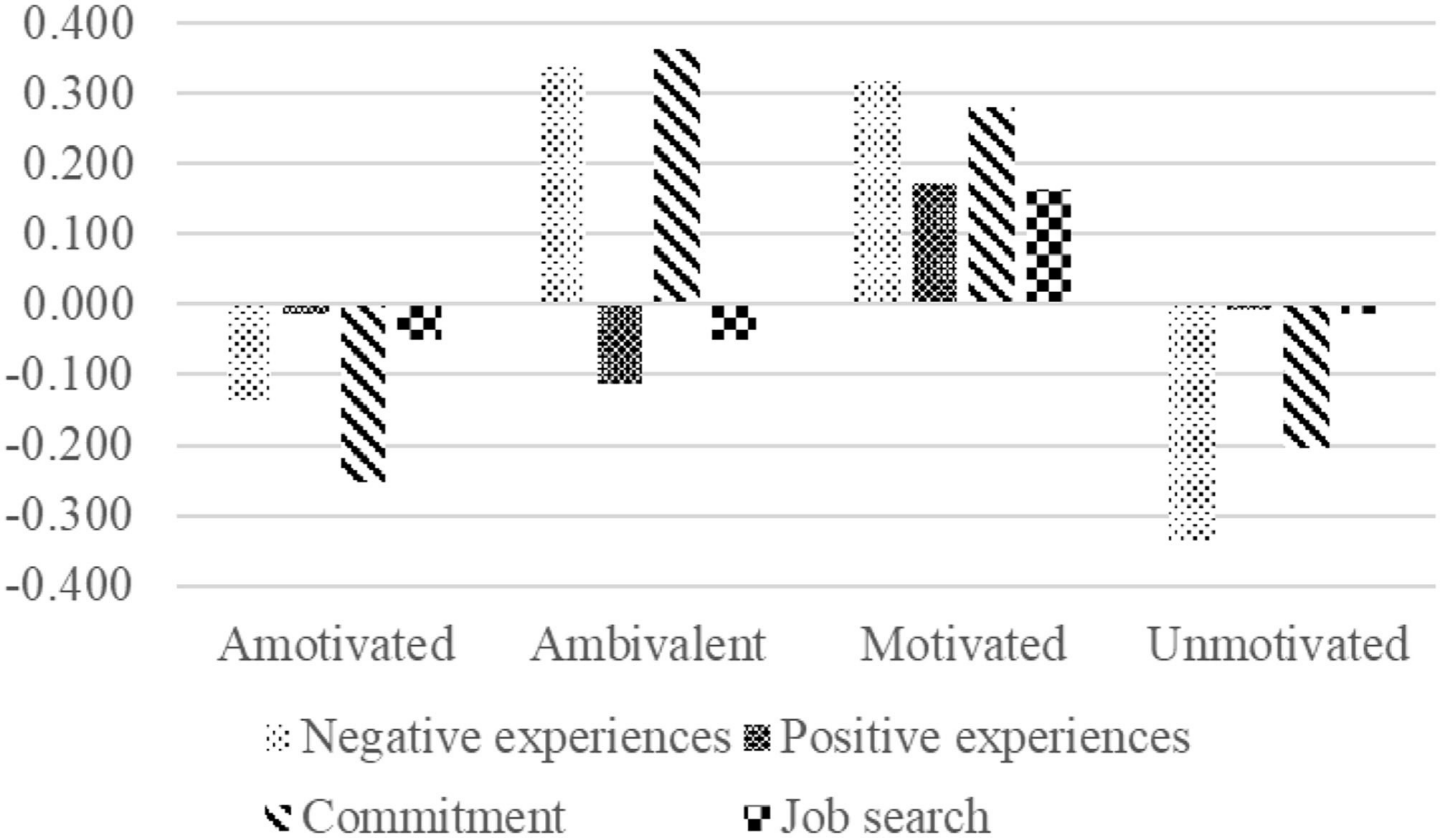
Negative experiences, positive experiences, employment commitment, and job search intensity as a function of membership of the motivational profiles.

We then proceeded by testing the associations between the motivational profiles and the well-being, attitudes, and behavior of the unemployed and tested Hypotheses 2 to 4. As displayed in the lower half of Table 3 and Figure 3, the BCH analysis indicated significant differences for each of the four outcomes.

According to Hypothesis 2, profiles with higher levels of amotivation should report the highest levels of negative experiences and the lowest levels of positive experiences, followed by profiles with higher levels of controlled motivation. In contrast, autonomously motivated profiles should report the lowest levels of negative experiences and the highest levels of positive experiences. Our results, however, showed that the unemployed in the motivated and the ambivalent groups reported the highest (but not significantly different) levels of negative experiences, followed by the amotivated group and, lastly, the unmotivated group. Apart from the results of the ambivalent group, the findings contradicted our expectations as set out in Hypothesis 2. Our results showed that the motivated group reported significantly more positive experiences, followed by the amotivated and unmotivated groups (who did not differ significantly from each other). The ambivalent group reported the least positive experiences. Therefore, the finding provided support for Hypothesis 2.

According to Hypothesis 3, we expected that profiles with higher levels of amotivation should report the lowest levels of commitment, followed by profiles with higher levels of controlled motivation. In contrast, autonomously motivated profiles should report the highest levels of commitment. Our results indicated that the motivated and the ambivalent groups were equally committed, but more committed than the unmotivated and amotivated groups (who did not differ significantly from each other). This result provided support for Hypothesis 3.

According to Hypothesis 4, we expected that profiles with higher levels of amotivation should then report the lowest levels of job search, followed by profiles with controlled motivation. In contrast, autonomously motivated profiles should report the highest levels of job search. Our results indicated that only the motivated reported significantly higher job search intensity. This result provided some support for Hypothesis 4.

## Discussion

This study aimed to examine the motivational functioning of the unemployed by exploring their profiles based on their different types of motivation (as mentioned in SDT) to look for a job and the associated affective experiences, attitudes, and job search behavior. This investigation would allow us to gain more theoretical knowledge on the associations of the combinations of the types of motivation (rather than using them as separate variables in the analysis) and provide practice with more ecologically valid suggestions on how to help the unemployed.

The different types of motivation combined naturally into four different groups. We first found an *amotivated profile* similar to the amotivated profiles previously reported in the sports and work contexts (13, 16, 59) and was the largest group (45.15%) in this sample. This profile reflects our reasoning that the unemployed might not search (or might search with less effort for different reasons) for a job in a discouraging context. Second, another profile with high levels of amotivation, that is, the *ambivalent*, was found. This profile is unique as profile research in other contexts typically reports a profile characterized by high levels of amotivation combined with low levels of other types of motivation (13, 20, 59), rather than the high levels of other types of motivation as found among the unemployed. This profile clearly illustrates that amotivation can manifest together with motivation in the context of unemployment. Some unemployed may, thus, feel inhibited by their environment or their own characteristics, but are still motivated to search for all kinds of different reasons. Together, these two profiles represented almost two-thirds of the sample, which is significantly more than the number of amotivated profiles typically reported in the literature (13, 59).

Alongside the profiles where amotivation played an important role, we also found a more *motivated* profile (only 18.60% of the sample). This profile aligns with previous profile research (59–61) and shows that behavior could be multi-motivated (9). Unemployed individuals may thus search for a job for various reasons simultaneously, and they may not have either controlled motivation or autonomous motivation. This profile clearly illustrates why variable-centered research (62) runs short in providing complete insights into people's motivation and the consequences of the interplay between various types of motivation.

Lastly, the results indicated an *unmotivated* profile (19.52% of the sample). The existence of this profile again contradicts most previous profile research (63–65), in which high(er) levels of amotivation combined with low levels of the other types of motivational regulation to form the unmotivated group. However, it corresponds somewhat to a profile identified by Abós et al. (66), which they labeled the low motivation group. As the opposite of the ambivalent group, this finding confirmed that even in the absence of barriers (resulting in amotivation), some unemployed individuals might still not be motivated to search for a job. It might be that these individuals had adapted to unemployment (24, 25) and/or had withdrawn from the labor market (67, 68).

In general, the amotivated were psychologically somewhat well (i.e., they had few negative experiences). Still, economically, they were less ideal as they displayed little commitment to employment and were not searching that intensely for a job. So, going through the motions of job search whilst not being compelled to or seeing its value protects the individual somewhat psychologically but means that one withdraws from the labor market. Similarly, the unmotivated may have experienced the least negative experiences, but not even going through the motions, not being compelled to, and seeing little to no value in searching for a job, was highly detrimental for labor market participation. The negative attitudinal and behavioral implications are in line with SDT and previous research where poor quality and low quantity profiles had worse outcomes than high quantity and good quality profiles (16, 28). Although the attitudinal and behavioral outcomes are in line with SDT, the finding that amotivation could associate with well-being is a challenging new finding in light of SDT. Still, the somewhat better psychological outcomes for the unmotivated than the amotivated profiles align with SDT and previous profile research, where profiles with high levels of amotivation are usually associated with more negative outcomes than those with low motivation (59, 61). This finding is not surprising as engaging in a job search, faced with seemingly insurmountable barriers, likely has more negative affective consequences for the unemployed than not engaging in the job search process (especially when combined with more controlled and less identified reasons as was the case for the amotivated).

In contrast, the ambivalent (and more so the motivated) group was economically more ideal. Both groups reported relatively high levels of commitment to employment, and the motivated also reported more job search intensity than all the other groups. So, being motivated to search for a job (regardless of the reason) fosters more positive attitudes and job search behavior (especially in the absence of amotivation). However, both these profiles suffered more psychologically (i.e., the highest levels of negative experiences) than their a- or unmotivated counterparts. These results may not be surprising. Studies have highlighted that mixed profiles may be more beneficial for performance (28, 69) than well-being (28, 70). Both profiles reported more controlled motivation than identified regulation, with the former being relatively beneficial for performance, yet detrimental for well-being (62). Motivated profiles also generally have more favorable attitudes (13, 70).

The motivated, furthermore, reported (the most) positive affective experiences, whereas, the ambivalent reported the least positive experiences. Experiencing unemployment as both negative and positive may be unusual, but is not entirely uncommon [see (24, 25)]. Again, our findings showed the detrimental effect of perceiving the environment as constraining while being motivated to find a job. SDT proposes that, for optimal well-being, one should be able to freely engage in an action in line with what one wants and supported by the environment (71). In addition to the absence of amotivation, the more “balanced” experience (of external and introjected vs identified regulation) evident among the motivated provides more opportunities to report positive affective experiences and intensified job search efforts.

The study makes two important contributions to research. First, the nature and prevalence of the respective profiles reflected the frustrating impact of the environment on motivation and/or the adaptation of the unemployed who developed these profiles to cope with their situation. There was no profile with optimal motivation (i.e., a profile characterized by high levels of autonomous motivation and low levels of controlled motivation and amotivation), and more than half of the sample reported high levels of amotivation. Amotivation is not always included in profile studies, but the latter finding illustrates the importance of including amotivation, especially in investigations conducted in discouraging environments. Our findings allowed us to generalize some of the previous research in both the work (13) and high school educational contexts (17, 72), which did not obtain highly self-determined profiles, but also supported the contextual influences argument to the extent that one peripheral profile (i.e., ambivalent) unique to this context emerged. SDT is a “positive” theory that essentially assumes that people inherently strive for self-determination (73). Still, in this context, one has to critically reflect on the manifestation of self-determination.

Second, the combination of different reasons for job search into motivational profiles illustrated that—in combination—the different types of motivation might show unexpected associations with an individual's well-being, attitudes, and behaviors. Being motivated or ambivalent is detrimental to well-being, but conducive to employment commitment. Being motivated is also conducive to job search intensity. These findings extend variable-centered findings illustrating that while amotivation and controlled motivation to look for a job are important predictors of negative experiences, attitudes, and behavior, searching for a job because you truly value the activity (i.e., identified regulation) may alleviate the burden of unemployment (5–7). Our results challenge these findings because the results suggest that the negative impact of amotivation and controlled motivation co-depends on the level of identified regulation within a person. For example, being motivated (i.e., having a profile with high levels of external and introjected regulation combined with high levels of introjected regulation) is beneficial for job search intensity and positive affective experiences. Also, being ambivalent (i.e., having a profile with high levels of all types of [a]motivation) results in positive attitudes and supports job search efforts.

### Limitations and Suggestions for Future Research

Although the study made a notable contribution to motivation and unemployment research using sophisticated statistical methods, some limitations are worth mentioning. The cross-sectional design made it impossible to reach conclusions regarding the direction of the associations between the profiles and the “dependent” variables and the stability of profile membership over time. To understand the causality of the relations (13) and the dynamics of the profiles (or profile membership over time (34, 74, 75), future longitudinal research is warranted. In this regard, we advise that researchers follow the recommendations of Spector (76) when designing longitudinal studies.

The strength of the study lies in the novel context in which it investigated profiles. Although the sample was mostly typical of the unemployed in SA, future research in different samples of the unemployed (in SA and elsewhere) could strengthen the validity (and generalizability) of these profiles. In line with sample recommendations, even though our sample was quite large and exceeded the recommendations for the minimum sample size (*n* = 500) for LPA (51), we cannot discount the possibility that a larger group of participants would not have yielded an additional (e.g., self-determined) group that might exist in the population. Therefore, additional and larger samples are needed to (dis)confirm the existence of such a profile.

Other limitations include the sampling technique, scales that needed to be adapted, the questionnaire that was kept simple (i.e., a three-item response scale), the use of an interviewer, and no follow-up in terms of long-term effects of finding employment. Although we took different avenues to sample, if we genuinely want to understand this problem, we must also examine the extreme groups (that are less accessible to researchers) that do not have access to benefits or even a sustainable livelihood during unemployment. Other recommendations include expanding the scale response options (within reasonable limits), validating the adapted scales in more samples, possibly excluding the interviewers for more genuine responses, and measuring whether the participants found jobs. Future research may also include other factors that are important in the psychological impact of unemployment to enhance our understanding of the psychological burden of unemployment [see (3, 77)]. The inclusion of psychological need satisfaction and need frustration would also provide important empirical evidence regarding the ability of psychological needs to support some of our explanations [see (8)].

Differentiating between different types of amotivation is perhaps not valuable for person-centered research in the unemployment context; the different types of amotivation tended to covary in this sample. However, it was interesting from a variable-centered perspective, as although the two types of amotivation were highly related, they yielded different relations with the other types of motivation and the outcomes. Personal amotivation was more strongly related to identification (i.e., seeing personal barriers also associated with how important something could be both in terms of identification and commitment) than structural amotivation. This could be partly adaptive, but also maladaptive, depending on whether these personal characteristics were malleable or not. However, it was primarily structural amotivation that linked to search behavior and high negative and low positive affective experiences. Therefore, our results open up new avenues for research in which amotivation is treated in a more nuanced way (i.e., multidimensional as opposed to unidimensional), similar to the other types of motivations.

### Practical Implications

According to the World Health Organization (WHO), mental health is a combination of the absence of mental disorders and the presence of mental health and well-being (78). Consequently, public mental health focuses on the prevention of mental disorders as well as mental health promotion by means of policies, governance, and organization (79, 80) at the population level (80). Given the prevalence of unemployment (especially in a non-WEIRD [Western, educated, industrialized, rich, and democratic] context such as South Africa) and the negative implications of unemployment for mental health, policy-makers should prioritize the development of evidence-based remedial and proactive policies and practices that guide (re-) employment, as well as promote the mental health of those that remains unemployed. Motivational profiles can enhance both accessibility and comprehension of motivation research by practitioners and policy-makers when information is presented regarding an individual's functioning, rather than the functioning of a variable (81). Jahoda (82) highlighted the importance of motivation in unemployment almost four decades ago, and meta-analytic results support the value of enhancing motivation through job search interventions (83). If we can tell them about unemployed people's (a)motivation, they can design interventions for unique groups. Several recommendations can be made. First, unemployment counselors should think about different people when they counsel the unemployed because they are not one homogeneous group. Second, as the motivated is the more adaptive profile, with presumably better prospects of finding employment, interventions should concentrate on facilitating (re-)employment among those with this profile. Third, interventions should aim to reduce the number of individuals in the ambivalent profile, which is the least psychologically adaptive profile—for example, by increasing one's coping with barriers or removing the barriers. Lastly, although the amotivated and unmotivated are not less desirable from a psychological perspective, they are from an economic perspective. They have less commitment to employment, and if left unattended, their search behavior will likely decrease to minimize the discrepancy between values and behavior. Interventions should, however, not blindly focus on “activating” these two groups, as they can easily turn into ambivalent rather than motivated. Following the caution not to blindly “activate” them, job creation and entrepreneurial interventions are recommended in conjunction with psychological interventions.

## Conclusion

The results of this study indicated the existence of different motivational profiles, consisting of different levels and types of motivational regulation. Furthermore, it was found that these profiles induced different outcomes—with the more motivated profile being more beneficial—providing support for the construct validity of these profiles. Interpretation highlighted that affective, attitudinal, and behavioral consequences depended on the combination of motivational types rather than the types in isolation. Employment counselors and policymakers should tailor unemployment interventions to suit the needs and expectations of the different types of unemployed to enhance the effectiveness of, and satisfaction with, these interventions.

## Data Availability Statement

The raw data supporting the conclusions of this article will be made available by the authors, without undue reservation.

## Ethics Statement

The studies involving human participants were reviewed and approved by Humanities and Health Research Ethics Committee (HHREC), North-West University and the Social and Societal Ethics Committee (SMEC), KU Leuven. The participants provided their written informed consent to participate in this study.

## Author Contributions

LV took the lead in conceptualizing, writing the article, and collected and analyzed the data. AV, SR, and HD acted as additional writers and reviewed the article. All authors contributed to the article and approved the submitted version.

## Conflict of Interest

The authors declare that the research was conducted in the absence of any commercial or financial relationships that could be construed as a potential conflict of interest.

## Publisher's Note

All claims expressed in this article are solely those of the authors and do not necessarily represent those of their affiliated organizations, or those of the publisher, the editors and the reviewers. Any product that may be evaluated in this article, or claim that may be made by its manufacturer, is not guaranteed or endorsed by the publisher.

## Footnotes

^1^The Supplementary Material contains a comprehensive overview of the definition and operationalization of motivation for the current study.

^2^The instrument was adapted in the current study through the elimination of items that were not relevant in South Africa (e.g., “because I find it enjoyable to explore the job market and search for jobs for which I am qualified”), and some items were adapted (e.g., “because I am too poorly educated to be accepted by an employer”). The amotivation items were developed guided by research on discouraged job seekers in which the barriers that unemployed people experienced discouraged them from seeking work. Here, it was argued that unemployed people were mostly discouraged by poverty, high unemployment rates, own duration of unemployment, the cost of the job search, and lack of education, to name a few (33).

^3^Model improvement included deleting one item from the importance of work scale (“People do not have to work as such to be constructively occupied”, insignificant factor loading). Two correlations between the residuals of items were allowed: “My self-worth has decreased” and “I have lost my self-confidence” (MI = 81.92) due to the theoretical overlap and “Searched for advertisements in newspapers and weeklies” and “Searched for advertisements on the internet (e.g. job or organizational websites) or social media (e.g. Facebook, LinkedIn)” (MI = 62.61) due to similar prompts.

## References

[1] Arora T Grey I Östlundh L Lam KBH Omar OM Arnone D. The prevalence of psychological consequences of COVID-19: a systematic review and meta-analysis of observational studies. J Health Psychol. (2020) 27:805–24. 10.1177/1359105320966639 33118376

[2] Du Toit M De Witte H Rothmann S Van Den Broeck A. Contextual factors and the experience of unemployment: a review of qualitative studies. S Afr J Econ Manage Sci. (2018) 21:a2083. 10.4102/sajems.v21i1.2083

[3] Wanberg CR. The individual experience of unemployment. Annu Rev Psychol. (2012) 63:369–96. 10.1146/annurev-psych-120710-100500 21721936

[4] Vansteenkiste M Van den Broeck A. Understanding the motivational dynamics among unemployed individuals: refreshing insights for the self-determination theory perspective. In: Klehe U van Hooft EAJ editors. The Oxford Handbook of Job Loss and Job Search. Oxford, UK: Oxford University Press (2018). pp. 159–79.Oxford, UK: Oxford University Press

[5] Vansteenkiste M Lens W De Witte S De Witte H Deci EL. The “why” and “why not” of job search behaviour: their relation to searching, unemployment experience, and well-being. Eur J Soc Psychol. (2004) 34:345–63. 10.1002/ejsp.202

[6] Vansteenkiste M Lens W De Witte H Feather NT. Understanding unemployed people's job search behaviour, unemployment experience and well-being: a comparison of expectancy-value theory and self-determination theory. Br J Social Psychol. (2005) 44:268–87. 10.1348/014466604X17641 16004649

[7] Koen J Van Vianen AEM Van Hooft EAJ Klehe U-T. How experienced autonomy can improve job seekers' motivation, job search, and chance of finding reemployment. J Vocational Behav. (2016) 95–96:31–44. 10.1016/j.jvb.2016.07.003

[8] Van der Vaart L Van den Broeck A Rothmann S De Witte H. Experiences, attitudes, and behaviors of the unemployed: the role of motivation and psychological needs. Psychol Rep. (2020) 23:1117–44. 10.1177/0033294119849020 31094660

[9] Vansteenkiste M Mouratidis A. Emerging trends and future directions for the field of motivation psychology: a special issue in honor of Prof. Dr Willy Lens. Psychol Belg. (2016) 56:317–41. 10.5334/pb.354 30479443 PMC5854157

[10] Fernet C Litalien D Morin AJS Austin S Gagné M Lavoie-Tremblay M. On the temporal stability of self-determined work motivation profiles: a latent transition analysis. Eur J Work Organ Psychol. (2020) 29:49–63. 10.1080/1359432X.2019.1688301

[11] Tóth-Király I Morin AJS Bothe B Rigó A Orosz G. Toward an improved understanding of work motivation profiles. Appl Psychol Int Rev. (2021) 70:986–1017. 10.1111/apps.12256

[12] Gillet N Fouquereau E Vallerand RJ Abraham J Colombat P. The role of workers' motivational profiles in affective and organizational factors. J Happiness Stud. (2018) 19:1151–74. 10.1007/s10902-017-9867-9

[13] Howard J Gagné M Morin AJS Van den Broeck A. Motivation profiles at work: a self-determination theory approach. J Vocational Behav. (2016) 95–96:74–89. 10.1016/j.jvb.2016.07.004

[14] Lv B Lv L Wang P Luo L. A person-centered investigation of math motivation and its correlates to math achievement in elementary students. J Pacific Rim Psychol. (2019) 13:e24. 10.1017/prp.2019.21

[15] Moran CM Diefendorff JM Kim TY Liu Z. A profile approach to self-determination theory motivations at work. J Vocat Behav. (2012) 81:354–63. 10.1016/j.jvb.2012.09.00231565008

[16] Tóth-Király I Amoura C Bothe B Orosz G Rigó A. Predictors and outcomes of core and peripheral sport motivation profiles: a person-centered study. J Sports Sci. (2020) 38:897–909. 10.1080/02640414.2020.1736765 32156190

[17] Tóth-Király I Morin AJS Litalien D Valuch M Bothe B Orosz G. Self-determined profiles of academic motivation. Motiv Emot. (2022) 46:152–170. 10.1007/s11031-021-09918-x

[18] Richards DK Pearson MR Field CA. Profiles of motivations for responsible drinking among college students: a self-determination theory perspective. Addict Behav. (2020) 111:106550. 10.1016/j.addbeh.2020.106550 32745942 PMC7839311

[19] Weske U Schott C. What motivates different groups of public employees working for Dutch municipalities? Combining autonomous and controlled types of motivation. Rev Public Pers Adm. (2018) 38:415–30. 10.1177/0734371X16671981

[20] Gillet N Morin AJS Ndiaye A Colombat P Fouquereau E. A test of work motivation profile similarity across four distinct samples of employees. J Occup Organ Psychol. (2020) 93:988–1030. 10.1111/joop.12322

[21] Graves LM Cullen KL Lester HF Ruderman MN Gentry WA. Managerial motivational profiles: composition, antecedents, and consequences. J Vocat Behav. (2015) 87:32–42. 10.1016/j.jvb.2014.12.002

[22] Meyer JP Morin AJS. A person-centered approach to commitment research: theory, research, and methodology. J Organ Behav. (2016) 37:584–612. 10.1002/job.2085

[23] Jahoda M. Employment and Unemployment: A Social-Psychological Analysis. Cambridge: Cambridge University Press (1982).

[24] Putter I Van Der Vaart L De Witte H Rothmann S Van Den Broeck A. Profiling the unemployed from selected communities in South Africa based on their experiences, commitment to employment, and job search behaviour. S Afr J Psychol. (2021) 51:533–46. 10.1177/0081246320978969

[25] Van der Vaart L De Witte H Van den Broeck A Rothmann S. A psychosocial typology of the unemployed in South Africa. S Afr J Psychol. (2018) 48:179–92. 10.1177/0081246317721600

[26] De Witte H Rothmann S Jackson LTB. The psychological consequences of unemployment in South Africa. S Afr J Econ Manag Sci. (2012) 15:235–52. 10.4102/sajems.v15i3.15326261909

[27] Warr P Cook JD Wall TD. Scales for the measurement of work attitudes and aspects of psychological well-being. J Occup Psychol. (1979) 52:129–48. 10.1111/j.2044-8325.1979.tb00448.x

[28] Levesque-Côté J Fernet C Morin AJS Austin S. On the motivational nature of authentic leadership practices: a latent profile analysis based on self-determination theory. Leadersh Organization Dev J. (2021) 42:178–94. 10.1108/LODJ-12-2019-0522

[29] McKee-Ryan F Song Z Wanberg CR Kinicki AJ. Psychological and physical well-being during unemployment: a meta-analytic study. J Appl Psychol. (2005) 90:53–76. 10.1037/0021-9010.90.1.53 15641890

[30] Maloma I. The role of downstream steel manufacturing co-operatives in job creation poverty alleviation in Boipatong (Master's thesis). North-West University, Vanderbijlpark, South Africa (2005). http://hdl.handle.net/10394/2362

[31] City of Johannesburg. Orange Farm: Beauty in the Land of the Poor. (2015). Available online at: http://www.joburg.org.za/index.php?option=com_contentandid=932andItemid=52

[32] De Kock F Kanjee A Foxcroft C. Cross-cultural test adaptation, translation, and tests in multiple languages. In: Foxcroft C Roodt G editors. Introduction to Psychological Assessment in the South African Context. 4th ed. Oxford, UK: Oxford University Press (2013). pp. 83–106.

[33] Kingdon GG Knight J. The measurement of unemployment when unemployment is high. Labour Econ. (2006) 13:291–315. 10.1016/j.labeco.2004.09.003

[34] De Witte H Hooge J Vanbelle E. Do the long-term unemployed adapt to unemployment? Rom J Appl Psychol. (2010) 12:8–14.

[35] Muthén B Muthén L. Mplus User's Guide. 8th ed. (1998–2021). Muthén and Muthén.

[36] Morin AJS Myers ND Lee S. Modern factor analytic techniques: bifactor models, exploratory structural equation modeling (ESEM) and bifactor-ESEM. In: Tenenbaum G Eklund RC editors. Handb Sport Psychol. 4th Edn. (2020). p. 1044–1073. 10.1002/9781119568124.ch51

[37] Howard JL Gagné M Morin AJS Forest J. Using bifactor exploratory structural equation modeling to test for a continuum structure of motivation. J Manag. (2018) 44:2638–664. 10.1177/014920631664565330909847

[38] Howard JL Gagné M Morin AJS. Putting the pieces together: reviewing the structural conceptualization of motivation within SDT. Motiv Emot. (2020) 44:846–61. 10.1007/s11031-020-09838-2

[39] Howard JL Gagné M Van Den Broeck A Guay F Chatzisarantis N Ntoumanis N. A review and empirical comparison of motivation scoring methods: an application to self-determination theory. Motiv Emot. (2020) 44:534–48. 10.1007/s11031-020-09831-9

[40] Kline RB. Principles and Practice of Structural Equation Modeling. 4th ed. New York: The Guilford Press (2016).

[41] Van Zyl LE Ten Klooster PM. Exploratory structural equation modelling: practical guidelines and tutorial with a convenient online tool for Mplus. Front Psychiatry. (2022) 12:e795672. 10.3389/fpsyt.2021.795672 35069293 PMC8779472

[42] Gadermann AM Guhn M Zumbo BD. Estimating ordinal reliability for likert-type and ordinal item response data: a conceptual, empirical, and practical guide. Pract Assess Res Eval. (2012) 17:1–13. 10.7275/n560-j767

[43] Morin AJS Meyer JP Creusier J Biétry F. Multiple-group analysis of similarity in latent profile solutions. Organ Res Methods. (2016) 19:231–54. 10.1177/1094428115621148

[44] Skrondal A Laake P. Regression among factor scores. Psychometrika. (2001) 66:563–76. 10.1007/BF02296196

[45] Muthén B Muthén LK. Integrating person-centered and variable-centered analyses: growth mixture modeling with latent trajectory classes. Alcohol Clin Exp Res. (2000) 24:882–91. 10.1111/j.1530-0277.2000.tb02070.x 10888079

[46] Morin AJS Bujacz A Gagné M. Person-centered methodologies in the organizational sciences: introduction to the feature topic. Organ Res Methods. (2018) 21:803–13. 10.1177/1094428118773856

[47] Woo SE Jebb AT Tay L Parrigon S. Putting the “person” in the center: review and synthesis of person-centered approaches and methods in organizational science. Organ Res Methods. (2018) 21:814–45. 10.1177/1094428117752467

[48] Wang J Wang X. Structural Equation Modeling. 2nd ed. Hoboken: Wiley (2020). 10.1002/9781119422730

[49] Tofighi D Enders CK. Identifying the correct number of classes in growth mixture models. In: Hancock GR Samuelsen KM editors. Advances in Latent Variable Mixture Models. Charlotte: Information Age Publishing (2008). pp. 317–341.

[50] Nylund KL Asparouhov T Muthén BO. Deciding on the number of classes in latent class analysis and growth mixture modeling: A Monte Carlo simulation study. Struct. Equ. Model. Multidiscip J. (2007) 14:535–69. 10.1080/10705510701575396

[51] Tein J Coxe S Cham H. Statistical power to detect the correct number of classes in latent profile analysis. Struct Equ Modeling. (2013) 20:640–57. 10.1080/10705511.2013.824781 24489457 PMC3904803

[52] Bakk Z Vermunt JK. Robustness of stepwise latent class modeling with continuous distal outcomes. Struct Equ Model. (2016) 23:20–31. 10.1080/10705511.2014.95510431619843

[53] Asparouhov T Muthén B. Auxiliary Variables in Mixture Modeling: Using the Bch Method in Mplus to Estimate a Distal Outcome Model an Arbitrary Secondary Model. (2021). Available online at: https://www.statmodel.com/examples/webnotes/webnote21.pdf

[54] Marsh HW Lüdtke O Trautwein U Morin AJS. Classical latent profile analysis of academic self-concept dimensions: synergy of person- and variable-centered approaches to theoretical models of self-concept. Struct Equ Model. (2009) 16:191–225. 10.1080/10705510902751010

[55] Nagin D. Group-Based Modeling of Development. Harvard: Harvard University Press (2005). 10.4159/9780674041318

[56] Clark SL. Mixture modeling with behavioral data (Doctoral dissertation). Available from ProQuest Dissertations and Theses database. (UMI No. 3405665) (2010)

[57] Field A. Discovering Statistics Using IBM SPSS Statistics. 4th ed. USA: Sage (2013).

[58] Cohen J. Statistical Power Analysis for the Behavioral Sciences. 2nd ed. Hillsdale, NJ: Lawrence Erlbaum (1988)

[59] Gustafsson H Carlin M Podlog L Stenling A Lindwall M. Motivational profiles and burnout in elite athletes: a person-centered approach. Psychol Sport Exerc. (2018) 35:118–25. 10.1016/j.psychsport.2017.11.009

[60] Abós Á Haerens L Sevil J Aelterman N García-González L. Teachers' motivation in relation to their psychological functioning and interpersonal style: a variable- and person-centered approach. Teach Teach Educ. (2018) 74:21–34. 10.1016/j.tate.2018.04.010

[61] Franco E Coterón J Gómez V Spray CM. A person-centred approach to understanding dark-side antecedents and students' outcomes associated with physical education teachers' motivation. Psychol Sport Exerc. (2020) 57:Article 102021. 10.1016/j.psychsport.2021.102021

[62] Van den Broeck A Howard J Van Vaerenbergh Y Leroy H Gagné M. Beyond intrinsic and extrinsic motivation: a meta-analysis on self-determination theory's multidimensional conceptualization of work motivation. Organ Psychol Rev. (2021) 11:240–73. 10.1177/20413866211006173

[63] Kong LC Liu WC. Understanding motivational profiles of high-ability female students from a Singapore secondary school: a self-determination approach. Asia Pac Educ Res. (2020) 29:529–39. 10.1007/s40299-020-00504-2

[64] Litalien D Gillet N Gagné M Ratelle CF Morin AJS. Self-determined motivation profiles among undergraduate students: a robust test of profile similarity as a function of gender and age. Learn Individ Differ. (2019) 70:39–52. 10.1016/j.lindif.2019.01.005

[65] Sass W Pauw J Donche V Petegem P. “Why (should) I do something for the environment?” Profiles of flemish adolescents' motivation toward the environment. Sustainability. (2018) 10:2579. 10.3390/su10072579

[66] Abós Á Haerens L Sevil-Serrano J Morbée S Julián JA García-González L. Does the level of motivation of physical education teachers matter in terms of job satisfaction and emotional exhaustion? a person-centered examination based on self-determination theory. Int J Environ Res Public Health. (2019) 16:2839. 10.3390/ijerph16162839 31398923 PMC6720261

[67] De Witte H. Tussen optimisten en teruggetrokken: Een empirisch onderzoek naar het psychosociaal profiel van langdurig werklozen en deelnemers aan de Weer-Werkactie in Vlaanderen [Between optimism and withdrawn: An empirical investigation into the psychosocial profile of the long-term unemployed]. KU Leuven, Hoger Instituut voor de Arbeid (1992).

[68] Statistics South Africa. Quarterly Labour Force Survey: Quarter 2, 2021. (2021). Available online at: https://www.statssa.gov.za/publications/P0211/P02112ndQuarter2021.pdf

[69] Chen C Zhang J Gilal FG. Composition of motivation profiles at work using latent analysis: theory and evidence. Psychol Res Behav Manag. (2019) 12:811–24. 10.2147/PRBM.S210830 31565008 PMC6731976

[70] Van den Broeck A Lens W De Witte H Van Coillie H. Unraveling the importance of the quantity and the quality of workers' motivation for well-being: a person-centered perspective. J Vocat Behav. (2013) 82:69–78. 10.1016/j.jvb.2012.11.005

[71] Deci EL Ryan RM. Motivation, personality, and development within embedded social contexts: an overview of self-determination theory. In: Ryan RM editor. Oxford Handbook of Human Motivation. Oxford, UK: Oxford University Press (2012). pp. 85–107. 10.1093/oxfordhb/9780195399820.013.0006

[72] Ratelle CF Guay F Vallerand RJ Larose S Senécal C. Autonomous, controlled, and amotivated types of academic motivation: a person-oriented analysis. J Educ Psychol. (2007) 99:734–46. 10.1037/0022-0663.99.4.734

[73] Sheldon KM. What's positive about positive psychology: reducing value-bias and enhancing integration within the field. In: Sheldon KM Kashdan TB Steger MF, editors. Designing Positive Psychology: Taking Stock and Moving Forward. Oxford, UK: Oxford University Press (2011). pp. 421-29. 10.1093/acprof:oso/9780195373585.003.0028

[74] da Motta Veiga SP Gabriel AS. The role of self-determined motivation in job search: a dynamic approach. J Appl Psychol. (2016) 101:350–61. 10.1037/apl0000070 26595757

[75] Howard JL Morin AJS Gagné M. A longitudinal analysis of motivation profiles at work. Motiv Emot. (2021) 45:39–59. 10.1007/s11031-020-09852-4

[76] Spector PE. Do not cross me: optimizing the use of cross-sectional designs. J Bus Psychol. (2019) 34:125–37. 10.1007/s10869-018-09613-8

[77] Paul KI Moser K. Unemployment impairs mental health: Meta-analyses. J Vocat Behav. (2009) 74:264–82. 10.1016/j.jvb.2009.01.001

[78] World Health Organization. Mental Health: Strengthening our Response. (2018). Available online at: https://www.who.int/news-room/fact-sheets/detail/mental-health-strengthening-our-response

[79] Jané-Llopis E Anderson P Stewart-Brown S Weare K Wahlbeck K McDaid D. Reducing the silent burden of impaired mental health. J Health Commun. (2011) 16:59–74. 10.1080/10810730.2011.601153 21916714

[80] Wahlbeck K. Public mental health: the time is ripe for translation of evidence into practice. World Psychiatry. (2015) 14:36–42. 10.1002/wps.20178 25655149 PMC4329888

[81] Vansteenkiste M Sierens E Soenens B Luyckx K Lens W. Motivational profiles from a self-determination perspective: the quality of motivation matters. J Educ Psychol. (2009) 101:671–88. 10.1037/a0015083

[82] Jahoda M. Work, employment, and unemployment: values, theories, and approaches in social research. Am Psychol. (1981) 36:184–91. 10.1037/0003-066X.36.2.184

[83] Liu S Hang JL Wang M. Effectiveness of job search interventions: a meta-analytic review. Psychol Bull. (2014) 140:1009–41. 10.1037/a0035923 24588365

