# Supplementary material for: Motivational Profiles in Unemployment: A Self-Determination Perspective
Source: Front Public Health. 2022 Apr 29;10:870073. doi: 10.3389/fpubh.2022.870073 (PMC9099225; doi:10.3389/fpubh.2022.870073)
Supplement: Supplementary file 1 [file Data_Sheet_1.PDF]

Supplementary Material

1 Operationalization of Motivation in the Current Study

According to SDT, motivation to search for a job can range from amotivation to controlled and autonomous types of motivation. Unemployed people may be amotivated or lack the motivation to search for different reasons. For example, they may believe that they cannot effectively search for a job, or they may expect no positive outcome from their job search efforts (i.e., they cannot control the outcome and feel helpless) (Ryan & Deci, 2017). Alternatively, they may not act because they have no interest in searching, or they may actively resist (or defy) searching for a job (Ryan & Deci, 2017). Unemployed people may also experience personal or human capital barriers, such as a lack of education or insufficient funds to search for a job, and environmental or structural barriers, such as high unemployment rates or adverse geographic locations (Fourie, 2012; Kingdon & Knight, 2006). Accordingly, the current study operationalized amotivation in terms of personal amotivation and structural amotivation.

Personal amotivation reflects amotivation resulting from the expectation that one lacks the human capital, i.e., competence or capacity, to perform a certain behavior necessary to achieve the desired outcomes. For example, unemployed individuals who are personally amotivated do not know how to start looking for a job, are too tired to look for a job, or lack the financial resources to look for a job. Structural amotivation reflects amotivation resulting from the expectation that structural factors prevent positive outcomes, even if one engages in the desired behaviors. For example, the structurally amotivated unemployed are discouraged from searching because they do not see any available jobs or feel that the government does not support them in finding employment.

Amotivation is not always included in profile studies, and when included, it is treated as a unidimensional construct (Howard et al., 2016; Fernet et al., 2020). However, because of its importance in the context of unemployment, in order to capture the motivation of the unemployed, we advanced the necessity of including amotivation in a more nuanced way. First, due to structural and human capital barriers, the unemployed may see the probability of finding work as limited (Kingdon & Knight, 2006), which fuels amotivation. Second, different reasons for being amotivated may have different implications (Ryan & Deci, 2017). For example, Legault et al. (2006) report that academic performance is negatively predicted by ability and effort beliefs, but not significantly predicted by the value placed on the task or the characteristics of the task. In short, differentiating between different subtypes of amotivation is, thus, important to gain a nuanced understanding of motivational profiles present in the unemployment context and their outcomes.

Apart from not being motivated, according to SDT, the unemployed may also be motivated to search for a job and may have different reasons to do so as well (Vansteenkiste & Van den Broeck, 2018). First, unemployed individuals may search for a job to meet external demands or pressures in the form of gaining a reward or avoiding punishment, which can be material (i.e., money) or social (i.e., praise or criticism) in nature. This is labelled external regulation. Second, unemployed individuals may exert pressure on themselves to search for a job in the form of self-imposed guilt, feelings of shame, or the threat of feeling less worthy as a person. This is labelled introjected regulation (Ryan & Deci, 2017). External regulation and introjected regulation are two subtypes of controlled motivation, which entails the unemployed participating in an activity out of (external or internal) pressure (Vansteenkiste & Van den Broeck, 2018).

In contrast to searching due to (external or internal) pressure, unemployed individuals may search because they perceive employment as important or meaningful (Vansteenkiste & Van den Broeck, 2018). Rather than feeling pushed, they then accept the reason for searching as their own and volitionally engage in a job search. This is labelled identified regulation (Ryan & Deci, 2017). Note that the identified unemployed do not experience exploration of the job market as pleasant or enjoyable, as would be the case with intrinsic motivation (Van Hooft et al., 2013). Instead, identified regulation is a type of extrinsic motivation, but

whereas external and introjected types are controlled types of extrinsic motivation, identified regulation is considered an autonomous type of extrinsic motivation (Ryan & Deci, 2017). Note that we excluded intrinsic regulation. We argue that the unemployed may, at most, see the job search task as instrumental in achieving valued outcomes – i.e., finding employment – but will not regard the search activity itself as fun or interesting. The social context, which is a significant antecedent of motivational regulation (Deci & Ryan, 2012), does not allow for people to enjoy only looking for a job. Discussions with unemployed individuals in South Africa prior to this research confirmed this assumption.

2 Model Comparison

Table S1 contains the goodness-of-fit indices for each of the measurement models. Models 1-3 were the ICM-CFA versions of the measurement model. In Model 1, the five motivation factors were allowed to correlate. In Model 2, external and introjected regulations were collapsed into one factor (i.e., controlled motivation) due to the high correlation ($r = .90$) between the two factors. Model 3 was similar to Model 1, except that the items were also allowed to load directly onto a general (motivation) factor, resulting in a bifactor-CFA model. In this model, the factors were orthogonal (Morin et al., 2020). The remaining models (Models 4 and 5) were specified using ESEM principles. Target rotation was used for Model 4, whereas orthogonal rotation was used for Model 5. Models 4 and 5 only differ from Models 1 and 3 in that items were allowed to cross-load onto non-target factors, but these cross-loadings were targeted to be as close to zero as possible (Morin et al., 2020).

Model selection commenced with a comparison between the ICM-CFA (i.e., Model 1) and ESEM (i.e., Model 4) solutions as recommended by Morin et al. (2020) and Van Zyl and Ten Klooster (in press). Although the five-factor CFA and the ESEM solutions fitted the data well, the ESEM solution performed slightly better (i.e., higher CFI and TLI values, lower RMSEA and SRMR values; no overlapping CIs for the RMSEA value). Table S2 provides the factor loadings for the ICM-CFA and ESEM solutions. On average (and as expected), the factor loadings in the ICM-CFA ($|\lambda| = .35$ to $.93$; M = .75) solution were higher than those in ESEM ($|\lambda| = .30$ to $.98$; M = 0.68) solution. Regardless of the drop in factor loadings, the specific factors in the ESEM solution were well-defined and corresponded to the theoretically proposed relations between the items and the facets. In the ESEM solution, the target facet loadings were higher than the cross-loadings, which were generally very small[1] ($|\lambda| = -.29$ to 0.59; M = .04). Significant cross-loadings further supported the choice of the ESEM instead of the ICM-CFA model (Morin et al., 2016, 2020). When looking at the factor correlations reported in Table S3, they were smaller in the ESEM solution than in the ICM-CFA solution. They were also all in the expected direction, and most of them were significant. These various considerations (i.e., model fit, well-defined factors, and significant cross-loadings) led to the retention of the ESEM solution. The upper limit of the 95% CIs for the factor correlations ranged from -.15 to .74. Being below the .80 cut-off value, the result suggests that all subscales display sufficient discriminant validity (Ronkko & Cho, 2022).

An important question in selecting the optimal solution is whether the ESEM or the bifactor ESEM should be retained. Given their almost identical fit (and overlapping CIs for the RMSEA value), the more parsimonious model (i.e., ESEM solution) was retained. In addition to the fit indices, the factor loadings of external, introjected, and identified regulation were higher on their specific (target) factors than those on the general factor. This indicates that the general factor does not contribute to these items in the same way that it does for the two types of amotivation. This is especially the case for external regulation. This observation questions the validity of the bifactor-ESEM and supports the selection of the ESEM solution.

[1] A factor loading of .30 indicates a small cross-loading (simple structure), and those ≥ .30 indicate meaningful cross-loadings (complex structure) (Morin et al., 2020).

Table S1

Measurement Models (N = 867)

Model	χ^2	df	p-value	RMSEA	CFI	TLI	SRMR
Model 1: ICM-CFA (5-factors)	900.81	289	.00	.049 [.046, .053]	0.96	.95	.08
Model 2: ICM-CFA (2-factors)	959.81	293	.00	.051 [.048, .055]	0.96	.95	.08
Model 3: ICM-bifactor	3154.16	273	.00	.110 [.107, .114]	0.81	.77	.14
Model 4: ESEM (5-factors)	354.49	205	.00	.029 [.024, .034]	0.99	.98	.03
Model 5: Bifactor ESEM	275.89	184	.00	.024 [.018, .030]	0.99	.99	.03

Model 1: amotivation was specified as two latent variables, namely personal amotivation (8 items) and structural amotivation (5 items), together with external (5 items), introjected (5 items) and identified regulation (3 items) as separate but correlated factors. Model 2: similar to Model 1 except external and introjected regulations were modelled as a unidimensional latent variable, namely controlled motivation (10 items). Model 3: a bifactor model was specified with a general factor and five specific factors similar to Model 1. Model 4: ESEM version of Model 1 where factor loadings are allowed to cross-load onto non-target factors; Model 5: ESEM version of Model 3 where factor loadings of the specific factors are allowed to cross-load onto non-target specific factors as well as all items being allowed to load onto a general (motivation) factor.

Table S2

Standardized Factor Loadings for Independent and Exploratory Structural Equation Modelling

Items	ICM-CFA solution	ESEM solution				
	λ	Factor 1 (λ)	Factor 2 (λ)	Factor 3 (λ)	Factor 4 (λ)	Factor 5 (λ)
1. Structural amotivation						
Item 1	.786***	**0.617******	0.203***	0.082	-0.070	-0.093*
Item 2	.903***	**0.904******	-0.025	-0.059	0.100**	-0.063
Item 3	.914***	**0.975******	-0.068	-0.103**	0.063	0.007
Item 4	.864***	**0.787******	0.130**	-0.012	-0.037	0.138***
Item 5	.834***	**0.723******	0.165***	0.105*	-0.087*	0.074
2. Personal amotivation						
Item 1	.793***	-0.003	**0.800******	-0.006	0.008	-0.063
Item 2	.837***	0.061	**0.795******	-0.130**	0.080	0.023
Item 3	.739***	0.017	**0.677******	0.020	0.045	-0.255***
Item 4	.820***	0.412***	**0.427******	-0.024	0.087	0.092*
Item 5	.772***	0.177***	**0.599******	0.058	0.029	-0.096*
Item 6	.755***	-0.005	**0.804******	0.155**	-0.056	0.074
Item 7	.757***	-0.070	**0.843******	0.069	-0.024	0.004
Item 8	.675***	-0.127*	**0.815******	-0.114*	0.024	0.027
3. External regulation						
Item 1	.478***	-0.149*	0.132*	**0.460******	0.029	0.278***
Item 2	.740***	-0.054	0.014	**0.536******	0.320***	-0.042
Item 3	.349***	0.026	-0.053	**0.456******	-0.129*	0.257***
Item 4	.765***	0.124**	-0.067	**0.575******	0.337***	-0.286***
Item 5	.435***	-0.010	0.016	**0.337******	-0.040	0.454***
4. Introjected regulation						
Item 1	.733***	0.066	-0.035	0.585***	**0.302******	-0.146**
Item 2	.778***	-0.025	0.080	0.145**	**0.672******	0.050
Item 3	.614***	0.073	0.017	0.046	**0.501******	0.282***
Item 4	.816***	-0.062	0.112*	-0.142**	**0.946******	0.178***
Item 5	.749***	0.060	-0.030	0.058	**0.752******	-0.106*
5. Identified regulation						
Item 1	.830***	-0.023	-0.085	0.096	0.188***	**0.708******
Item 2	.932***	-0.046	-0.112	0.097	0.157**	**0.801******
Item 3	.804***	0.118*	-0.143*	0.136*	0.012	**0.775******

Notes. ESEM = Exploratory structural equation modelling; ICM-CFA = independent cluster model-confirmatory factor analysis; λ = Standardized factor loading; boldface indicates target ESEM factor loadings. * $p \leq .05$, ** $p \leq .01$, *** $p \leq .001$.

Table S3

Latent Factor Correlations from the Six-Factor ESEM (Under the Diagonal) and CFA (Over the Diagonal) Solutions

	1	2	3	4	5
1. Structural amotivation	-	.78***	.13**	.26***	-.08
2. Personal amotivation	0.70***	-	.05	.21***	-.30***
3. Extrinsic regulation	0.08	-0.03	-	.90***	.53***
4. Introjected regulation	0.23***	0.16*	0.58***	-	.40***
5. Identified regulation	-0.04	-0.25**	0.27**	0.12	-

Notes: M = Mean; SD = Standard deviation; Means and standard deviations estimated from scale scores indicated in brackets; omega reliability coefficients provided in brackets on the diagonal; * $p < .05$, ** $p < .01$, *** $p < .001$.

Table S4

Standardized Factor Loadings (λ) for Bifactor-Exploratory Structural Equation Modelling Solution

	General factor (λ)	Factor 1 (λ)	Factor 2 (λ)	Factor 3 (λ)	Factor 4 (λ)	Factor 5 (λ)
1. Structural amotivation						
Item 1	0.656***	**0.399*****	0.094*	0.059	-0.024	-0.068
Item 2	0.713***	**0.558*****	-0.048	-0.023	0.096**	0.005
Item 3	0.685***	**0.635*****	-0.023	-0.062	0.064*	0.055
Item 4	0.685***	**0.512*****	0.038	-0.003	-0.012	0.174***
Item 5	0.664***	**0.483*****	0.080	0.090*	-0.015	0.103*
2. Personal amotivation						
Item 1	0.818***	-0.128***	**0.135**	-0.033	-0.093*	-0.025
Item 2	0.872***	-0.103*	**0.116**	-0.170***	-0.053	0.093
Item 3	0.732***	-0.068	**0.184**	-0.032	-0.010	-0.237***
Item 4	0.742***	0.218***	**0.074**	-0.017	0.051	0.143**
Item 5	0.727***	0.081*	**0.199****	0.021	0.016	-0.082
Item 6	0.655***	0.082	**0.451*****	0.114**	0.000	0.002
Item 7	0.645***	0.053	**0.500*****	0.024	0.009	-0.100*
Item 8	0.581***	0.002	**0.476*****	-0.139**	-0.020	-0.084
3. External regulation						
Item 1	-0.084	0.026	0.195***	**0.474*****	0.225***	0.271***
Item 2	0.079	-0.018	-0.004	**0.563*****	0.500***	0.050
Item 3	-0.074	0.021	-0.057	**0.408*****	0.059	0.317***
Item 4	0.286***	-0.056	-0.239***	**0.552*****	0.492***	-0.081
Item 5	-0.124	0.101	0.107	**0.338*****	0.134**	0.453***
4. Introjected regulation						
Item 1	0.213**	-0.053	-0.175**	0.563***	**0.475*****	0.037
Item 2	0.226***	-0.026	-0.023	0.257***	**0.682*****	0.154***
Item 3	0.091	0.141**	0.088	0.170***	**0.528*****	0.305***
Item 4	0.201***	0.031	0.096*	0.067	**0.863*****	0.223***
Item 5	0.236***	0.018	-0.069	0.193***	**0.721*****	0.005
5. Identified regulation						
Item 1	-0.208**	0.068	0.004	0.173***	0.266***	**0.718*****
Item 2	-0.236**	-0.003	-0.097	0.155***	0.232***	**0.837*****
Item 3	-0.183*	0.130*	-0.066	0.177***	0.118**	**0.793*****

Notes. Boldface indicates target ESEM factor loadings. $^{*}p \leq .05$, $^{**}p \leq .01$, $^{***}p \leq .001$.

3 Associations Between Background Characteristics and Motivational Profiles

Due to the categorical nature of variables, associations between them were calculated using cross-tabulations (also known as contingency tables). Cross-tabulations are equivalent to correlations used to determine the relationships between continuous variables. Cramer's V (the equivalent of the rho coefficient used in continuous correlations) was computed to provide a measure of the strength of the association between the categorical variables (Field 2018). The following guidelines were used to determine the magnitude of the association: strong (>.50), moderate (.30 to .49), weak (.10 to .29) (Cohen, 1988).

Table S5

Background Characteristics by Motivational Profiles

	N (%)	Amotivated (Expected count)	Ambivalent (Expected count)	Motivated (Expected count)	Unmotivated (Expected count)
Age					
18-24	232 (27.00)	140 (107.00)	21 (37.50)	35 (42.20)	36 (45.10)
25-34	328 (38.20)	163 (151.20)	44 (53.10)	56 (59.90)	65 (63.80)
35-54	263 (30.60)	82 (121.20)	66 (42.60)	54 (48.10)	61 (51.10)
55-64	36 (4.20)	11 (16.60)	8 (5.80)	12 (6.60)	5 (7.00)
$\chi^2(df)$	$59.37(9)^{**}$				
Cramer's V	$.15^{**}$				
Education					
Below Grade 12	505 (58.70)	212 (231.10)	108 (83.30)	102 (92.10)	83 (98.50)
Grade 12	333 (38.70)	170 (152.40)	33 (54.90)	51 (60.70)	79 (65.00)
Above Grade 12	23 (2.70)	12 (10.50)	1 (3.80)	4 (4.20)	6 (4.50)
$\chi^2(df)$	$30.58(6)^{**}$				
Cramer's V	$.13^{**}$				
Marital status					
With partner	138 (16)	43(63.50)	22 (22.40)	31 (25.20)	42 (26.90)
Without partner	723 (84)	353 (332.50)	118 (117.60)	126 (131.80)	126 (141.10)
$\chi^2(df)$	$19.53(3)^{**}$				
Cramer's V	$.15^{**}$				

Table S5 (*continues…)*

	N (%)	Amotivated (Expected count)	Ambivalent (Expected count)	Motivated (Expected count)	Unmotivated (Expected count)
Living situation					
Living with parents or grandparents (or other family members)	435 (50.50)	212 (200.10)	55 (71.70)	88 (79.30)	80 (83.90)
Living alone	34 (9.30)	34 (36.80)	16 (13.20)	16 (14.60)	14 (15.40)
Living with children (single parent)	116 (13.50)	58 (53.40)	36 (19.10)	14 (21.20)	8 (22.40)
Living with partner that is unemployed	108 (12.50)	41 (49.70)	17 (17.80)	19 (19.70)	31 (20.80)
Living with partner that is employed	91 (10.60)	38 (41.90)	18 (15.00)	12 (16.60)	23 (17.50)
Other	31 (3.60)	13 (14.30)	0 (5.10)	8 (5.70)	10 (6.00)
χ^2(d*f*)	53.12**				
Cramer's V	.14**				
Area					
Boipatong	470 (54.20)	190 (216.30)	108 (77.50)	68 (85.10)	104 (91.10)
Orange Farm	397 (45.80)	209 (182.70)	35 (65.50)	89 (71.90)	64 (76.90)
χ^2(df)	44.67**				
Cramer's V	.23**				
Grants (claimed by others)					
No one	277 (33.90)	115 (127.80)	61 (47.10)	61 (49.50)	40 (52.60)
One	258 (31.60)	117 (119.10)	45 (43.90)	43 (46.10)	53 (48.90)
Two	153 (18.70)	84 (70.60)	16 (26.00)	16 (27.30)	37 (29.00)
More than three	129 (15.80)	61 (59.50)	17 (21.90)	26 (23.10)	25 (24.50)
χ^2(d*f*)	26.49*				
Cramer's V	.10*				

Notes. * $p < .01$; ** $p < .001$.

4 References

Cohen, J. (1988). *Statistical power analysis for the behavioral sciences* (2nd ed.). Lawrence Erlbaum.

Deci, E. L., & Ryan, R. M. (2012). Motivation, personality, and development within embedded social contexts: An overview of self-determination theory. In R. M. Ryan (Ed.), *Oxford handbook of human motivation* (pp. 85–107). Oxford University Press.

Fernet, C., Litalien, D., Morin, A. J. S., Austin, S., Gagné, M., Lavoie-Tremblay, M., & Forest, J. (2020). On the temporal stability of self-determined work motivation profiles: a latent transition analysis. *European Journal of Work and Organizational Psychology*, *29*(1), 49–63. https://doi.org/10.1080/1359432x.2019.1688301

Fourie, C. v. N. (2012). *The South African unemployment debate: Three worlds, three discourses?* http://www.carnegie3.org.za/papers/85_Fourie_The%20SA%20Unemployment%20debate%20-%20three%20worlds,%20three%20discourses.pdf

Howard, J., Gagné, M., Morin, A. J. S., & Van den Broeck, A. (2016). Motivation profiles at work: A self-determination theory approach. *Journal of Vocational Behavior, 95–96*, 74–89. https://doi.org/10.1016/j.jvb.2016.07.004

Kanfer, R., Wanberg, C. R., & Kantrowitz, T. M. (2001). Job search and employment: A personality-motivational analysis and meta-analytic review. *Journal of Applied Psychology, 86*(5), 837–855. https://doi.org/10.1037/0021-9010.86.5.837

Legault, L., Green-Demers, I., & Pelletier, L. G. (2006). Why do high school students lack motivation in the classroom? Toward an understanding of academic amotivation and the role of social support. *Journal of Educational Psychology, 98*(3), 567–582. https://doi.org/10.1037/0022-0663.98.3.567

Morin, A. J. S., Meyer, J. P., Creusier, J., & Biétry, F. (2016). Multiple-group analysis of similarity in latent profile solutions. *Organizational Research Methods, 19*(2), 231–254. https://doi.org/10.1177/1094428115621148

Morin, A. J. S., Myers, N. D., & Lee, S. (2020). Modern factor analytic techniques: Bifactor models, exploratory structural equation modeling (ESEM) and bifactor-ESEM. *Handbook of Sport Psychology, 1*, 1–36. https://doi.org/10.1002/9781119568124.ch51

Rönkkoö, M., & Cho, E. (2022). An updated guideline for assessing discriminant validity. *Organizational Research Methods, 25*(1), 6–14. https://doi.org/10.1177/1094428120968614

Ryan, R. M., & Deci, E. L. (2017). *Self-determination theory: Basic psychological needs in motivation, development, and wellness*. The Guilford Press.

Van Hooft, E. A. J., Wanberg, C. R., & Van Hoye, G. (2013). Moving beyond job search quantity: Towards a conceptualization and self-regulatory framework of job search quality. *Organizational Psychology Review, 3*(1), 3–40. https://doi.org/10.1177/2041386612456033

Vansteenkiste, M., & Van den Broeck, A. (2018). Understanding the motivational dynamics among unemployed individuals: Refreshing insights for the self-determination theory perspective. In U. Klehe & E. A. J. van Hooft (Eds.), *The Oxford handbook of job loss and job search* (pp. 159–179). Oxford University Press.
